# Dysregulated HPA axis during postnatal developmental stages in the BTBR *T^+^ Itpr3^tf^/*J mouse: A model of autism spectrum disorder

**DOI:** 10.1002/npr2.12508

**Published:** 2024-11-28

**Authors:** Nozomi Endo, Atsuo Hiraishi, Sayaka Goto, Hitoshi Nozu, Takayo Mannari‐Sasagawa, Noriko Horii‐Hayashi, Michiko Kitsuki, Mamiko Okuda, Manabu Makinodan, Mayumi Nishi

**Affiliations:** ^1^ Department of Anatomy and Cell Biology Nara Medical University Kashihara Japan; ^2^ Department of Pharmacology, School of Medicine Hyogo Medical University Nishinomiya Japan; ^3^ KYOUSEI Science Center for Life and Nature Nara Women's University Nara Japan; ^4^ Department of Psychiatry Nara Medical University Kashihara Japan

**Keywords:** autism spectrum disorder, early life stages, endocrinology, mouse model, stress responses

## Abstract

Autism spectrum disorder (ASD) is a neurodevelopmental disorder. Some children with ASD show enhanced cortisol response to stress. BTBR *T* ^+^ *Itpr3* ^*tf*^/J (BTBR) mice, an ASD model, display behavior consistent with the three diagnostic categories of ASD and exhibit an exaggerated response to stress in adulthood. However, it remains unclear how basal corticosterone levels change and how the hypothalamic–pituitary–adrenal axis responds to stress during the early life stages in BTBR mice. In this study, we found that basal corticosterone levels showed characteristic changes, peaking at weaning during postnatal development in both BTBR and control C57BL/6J (B6J) mice. Furthermore, we observed higher corticosterone and corticotropin‐releasing hormone levels in BTBR mice than in B6J mice following acute stress exposure during weaning; however, adrenocorticotropic hormone levels were lower in BTBR mice. Glucocorticoid receptor mRNA expression levels in the hippocampus and lateral septum after stress were higher in BTBR mice than in B6J mice. This study documented changes in corticosterone levels at baseline during postnatal development in mice and showed that BTBR mice exhibited disrupted stress responses at weaning.

## Abstract 2

During postnatal development, both BTBR *T* ^+^ *Itpr3tf*/J (BTBR) mice, a model of autism spectrum disorder (ASD), and control C57BL/6J mice exhibited characteristic changes in basal corticosterone levels, peaking at weaning. At weaning, BTBR mice showed dysregulated stress responses characterized by higher corticosterone and corticotropin‐releasing hormone levels but lower adrenocorticotropic hormone levels after acute stress exposure. Additionally, BTBR mice displayed altered expression of glucocorticoid receptor mRNA in specific brain regions, suggesting disrupted hypothalamic–pituitary–adrenal axis function at weaning in this ASD model.

## Graphical abstract

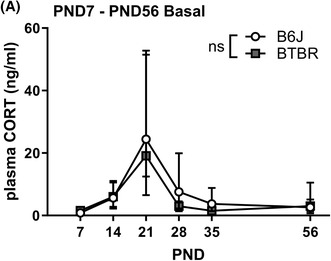

## 1 INTRODUCTION

Autism spectrum disorder (ASD) is one of the most commonly diagnosed neurodevelopmental disorders. According to the 2023 data from the Centers for Disease Control and Prevention (CDC), approximately one in 36 (2.8%) children have ASD.^1^ Earlier studies reported slightly lower rates, such as one in 45 children.^2^ The Diagnostic and Statistical Manual of Mental Disorders, Fifth Edition, Text Revision (DSM‐5‐TR), defines ASD using two core criteria: persistent deficits in social communication and social interaction and restricted, repetitive patterns of behavior, interests, or activities.^3^ In addition to these diagnostic criteria, individuals with ASD often exhibit other associated features such as sensory sensitivity, anxiety, and difficulties with executive functioning.^4^ Although the neurobiological mechanisms underlying ASD remain highly controversial, an enhanced stress response^5^, ^6^ and modified neuroendocrine function of the hypothalamic–pituitary–adrenal (HPA) axis^7^, ^8^ have been observed in individuals with ASD.

Abnormal stress response was detected in inbred BTBR *T* ^+^ *Itpr3* ^*tf*^/J mice (BTBR *T* ^+^ *tf*/J; referred to as BTBR henceforth), a widely used model of ASD. BTBR mice exhibit behavioral phenotypes resembling the core symptoms of ASD, such as reduced social interactions, unusual vocalizations, and increased repetitive behaviors.^9^, ^10^, ^11^, ^12^, ^13^ The BTBR strain has a unique genetic background derived from Black and Tan BRachyury lines. It carries several notable mutations, including a spontaneous mutation in *Itpr3* resulting in the “tuft hair” phenotype and a 25‐bp deletion in exon 6 of the *Disc1* gene. This *Disc1* deletion, also found in multiple strains of the 129 superfamily and other mouse strains, causes a frameshift, giving rise to a premature stop codon.^14^ A notable anatomical feature of BTBR mice is the complete agenesis of the corpus callosum, accompanied by a reduced hippocampal commissure.^15^ Neurochemically, these mice exhibit alterations in the serotonergic and dopaminergic systems^16^, ^17^ and differences in oxytocin signaling.^18^ They also show increased levels of pro‐inflammatory cytokines and altered microglial activation patterns,^19^ characteristics of interest in ASD research. We previously reported that BTBR mice exhibit lower activity levels in the dark phase and altered social behavior compared with control C57BL/6J mice (from now on referred to as B6J) under social housing conditions.^20^ Previous studies have reported that adult BTBR mice display increased baseline corticosterone (CORT) levels,^21^, ^22^, ^23^ and higher CORT levels are induced in adult BTBR mice after stress stimuli.^22^, ^24^ Gould et al. reported that social novelty elicited higher CORT levels in adolescent (5 weeks old) BTBR mice than in controls; however, at 16 weeks (adult), CORT levels induced by stress were comparable between BTBR and B6J mice.^25^ These results indicate abnormal function of the HPA axis in BTBR mice, which changes in an age‐dependent manner. ASD develops early; however, few studies have investigated the HPA axis during the early life stages in BTBR mice. In this study, we focused on the HPA axis during early postnatal developmental stages in BTBR mice using B6J mice as the control strain. This choice of control was consistent with that of numerous previous studies on BTBR mice, allowing for a better comparison and integration of our results with the existing body of research using BTBR mice as an ASD model.

Rodents undergo a specific period during postnatal development during which the HPA axis shows rapid regression, known as the stress hyporesponsive period. This period extends from postnatal day (PND) 2–14. During the early postnatal stages, maintaining low and stable levels of CORT is necessary for the normal growth and development of the central nervous system. The stress‐hyporesponsive period is hypothesized to be neuroprotective against stress‐induced exaggerated stimulation of glucocorticoid receptors (Nr3c1, GR).^26^, ^27^ However, most previous studies investigating the HPA axis in rodents focused on stress responses during later developmental stages; however, changes in basal CORT levels during early postnatal developmental stages remain unknown. Androgens are expressed at different levels during different developmental stages, and this time‐specific expression is essential for sexual differentiation in the brain.^28^, ^29^, ^30^ It is possible that the basal CORT level changes during the early life stages. However, it remains unclear how basal CORT levels change between early life stages and adulthood in rodents. In this study, we examined plasma CORT levels at basal levels from PND 7 to 56. Furthermore, we investigated plasma CORT and hormones that regulate CORT levels, namely corticotropin‐releasing hormone (CRH) and adrenocorticotropic hormone (ACTH), after acute stress exposure on PND 21 (weaning stage) to confirm whether abnormalities in the stress response were present in BTBR mice at this stage.

In addition to the endocrine experiments, we examined the stress response in BTBR mice at the gene expression level. CORT functions via Nr3c1 and the mineralocorticoid receptors (Nr3c2, MR). Dysregulation of the negative feedback of glucocorticoids via these two receptors can cause hyperactivity of the HPA axis.^31^ A previous study reported that *Nr3c1* mRNA levels in the CA1 region of the hippocampus were significantly higher in BTBR mice than in control mice.^23^ Brain Nr3c2 binds to CORT with a 10‐fold higher affinity than Nr3c1.^32^ Thus, at normal physiological concentrations of CORT, high‐affinity Nr3c2 is preferentially occupied, whereas Nr3c1s gradually become occupied by CORT when the levels are elevated by stress. Nr3c2‐ and Nr3c1‐mediated actions must be balanced to maintain metaplasticity and stress response behaviors.^33^ However, few studies have examined the expression of Nr3c1 and Nr3c2 in BTBR mice. Moreover, BTBR mice have severely reduced hippocampal commissure,^34^ a brain area that exhibits very high *Nr3c2* and *Nr3c1* mRNA expressions and regulates paraventricular hypothalamic nucleus (PVN) activity, the starting point of the HPA axis. In contrast, the PVN exhibited weak *Nr3c2* mRNA and strong *Nr3c1* expression. In this study, we investigated the expression levels and patterns of *Nr3c1* and *Nr3c2* mRNA in the hippocampus and PVN of BTBR mice using reverse transcriptase‐quantitative polymerase chain reaction (RT‐qPCR) and in situ hybridization (ISH), respectively. Meyza et al. examined the expression patterns of Fos, a neuronal activity marker, across many brain regions and reported that BTBR mice have a distinct pattern of neuronal responses to social stimuli compared with the control strain.^35^ We also examined *Fos* mRNA expression in the hippocampus and PVN at baseline and after acute stress.

Finally, we performed gene expression analysis in the lateral septum (LS), although it is generally not included in the HPA axis. The LS connects many brain regions, including the hippocampus and PVN, and serves as a key hub. Anatomically, the LS receives major inputs from the hippocampus and projects them to the regions involved in social and emotional processing, including the hypothalamus, amygdala, and ventral tegmental area.^36^, ^37^ The LS is involved in various brain functions, such as anxiety, fear, social behavior, and stress response.^38^, ^39^, ^40^, ^41^, ^42^ Consistent with its involvement in stress‐related functions, the LS expresses mRNA for *Nr3c1* and *Nr3c2*, which encode glucocorticoid and mineralocorticoid receptors, respectively.^43^, ^44^, ^45^ Given its central role in the regulation of social behavior, alterations in the LS function or connectivity could contribute to the social deficits observed in autism spectrum disorders.^15^ Because BTBR mice exhibit abnormal anatomical characteristics in the LS,^46^ we hypothesized that the expression of these stress‐related genes may be altered in this region. In this study, we investigated the expression patterns of stress‐related genes in the LS of BTBR mice to explore their potential associations with their unique phenotypes.

This study aimed to investigate the stress response in BTBR mice during the early developmental stages, focusing on the weaning period. Our objectives were to (1) characterize the developmental trajectory of basal CORT levels from PND 7 to 56; (2) examine the stress response on PND 21 by measuring plasma CORT, CRH, and ACTH levels after acute stress; and (3) analyze the expression of *Nr3c1*, *Nr3c2*, *Fos*, and *Arc* mRNA in the hippocampus, PVN, and LS under basal and stress conditions. This comprehensive approach seeks to elucidate the unique features of HPA axis function in BTBR mice on PND 21, potentially providing insights into stress‐related abnormalities in ASD.

## 2 MATERIALS AND METHODS

### 2.1 Mice

Male B6J (CLEA, Tokyo, Tokyo) and BTBR mice (Charles River Lab Inc., Yokohama, Japan) were housed in temperature‐ and humidity‐controlled animal facilities under a reverse light–dark cycle (lights on from 08:00 to 20:00). All animals were provided food and water ad libitum throughout the experiment. The day of birth was defined as PND 0. Pups were weaned on PND 21 and group‐housed with same‐sex siblings (2–5 pups/cage). All animal experimental protocols used in this study were approved by the Animal Care Committee of Nara Medical University.

### 2.2 Blood and brain sampling

Male BTBR and B6J mice on PND 7, 14, 21, 28, and 56 were immediately removed from their home cages between 10:00 and 12:30 and sacrificed by rapid decapitation. Trunk blood was collected in heparin‐containing tubes for CORT enzyme‐linked immunosorbent assay (ELISA) and in ethylenediaminetetraacetic acid‐containing tubes for CRH and ACTH ELISA. Plasma was obtained by centrifugation and stored at −80°C until the day of the assay. For mice on PND 21, the brains were removed immediately after decapitation, frozen on powdered dry ice, and stored at −80°C until further use. For each mouse group, 1–2 pups from each dam were used for each PND to reduce the effects of maternal care.

### 2.3 Stress exposure

On PND 21, male BTBR and B6J mice were immediately removed from their home cages between 10:00 and 12:00 h and subjected to restraint stress for 30 min. The hind and forepaws were attached to a board with paper tape for 30 min, during which time the mice were immobilized. After stress exposure, the mice were immediately sacrificed for sampling, as described above.

### 2.4 Enzyme‐linked immunosorbent assay (ELISA)

Plasma concentrations of CORT, CRH, and ACTH were measured using ELISA kits for CORT (Enzo Life Sciences, NY, USA; Cat. No. ADI 900–097), CRH (Phoenix Pharmaceuticals Inc., Belmont CA, USA; Cat. No. EK‐019‐06), and ACTH (MD Bioproducts, St Paul, MN, USA; Cat. No. M046006), respectively, according to the manufacturer's instructions. Standard curves were generated using the GraphPad Prism 7 software (GraphPad Software Inc., La Jolla, CA, USA). Specifically, we utilized the “Nonlinear Standard Curves” features to create standard curves from the concentration and optical density (OD) values of ELISA kit standards. Sample concentrations were interpolated from the OD values using the generated standard curves.

### 2.5 In situ hybridization histochemistry (ISH)

Coronal sections were cut at 10‐μm thickness using a cryostat (Leica 3050, Leica, Wetzlar, Germany). The coding regions of *Nr3c1* (positions 773–1616, 2854–3687, 4050–4858, and 5400–6123; GenBank accession No. NM_008173.4), *Nr3c2* (321–1090, 1239–1959, 3075–4183, and 4241–5100; GenBank no. NM_001083906.1), and *Fos* (223–985; GenBank no. NM_010234.3) were cloned into the pCR II‐TOPO vector (Invitrogen, La Jolla, CA, USA). Using linearized plasmids as templates, sense and antisense single‐stranded riboprobes were synthesized using a digoxigenin RNA labeling kit (Roche Diagnostics, Mannheim, Germany), and labeled nucleic acids were detected using the corresponding kit (Roche Diagnostics, Mannheim, Germany). ISH was performed as previously described^47^ with slight modification involving NBT/BCIP Stock Solution (Roche Diagnostics, Mannheim, Germany) for signal detection. Images of each section were acquired using a BZ‐X700 microscope (Keyence, Osaka, Japan).

### 2.6 Nissl staining

Nissl staining was performed on 10‐μm‐thick brain sections. The sections were first fixed in MildformR 20 N (20% neutral‐buffered formalin containing 7.5–8.5% formaldehyde; FUJIFILM Wako Pure Chemical Corporation, Osaka, Japan) for 10 min, followed by washing in phosphate‐buffered saline for 3 min, which was repeated thrice. Tissue dehydration was initiated by immersion in 70% ethanol for 5 min. The sections were then stained with a cresyl violet solution (Muto Pure Chemicals Co., Ltd., Tokyo, Japan) for 30 min. After staining, the sections were subjected to a graded ethanol series for differentiation and further dehydration: 70% ethanol for 3 min, 95% ethanol for 3 min, and 100% ethanol for 5 min. Clearing was achieved through two 10‐min immersions in Lemosol (95.0 + % limonene, a xylene substitute; FUJIFILM Wako Pure Chemical Corporation, Osaka, Japan). Finally, the sections were mounted using Entellan® New (Merck, Darmstadt, Germany) as a mounting medium. Images of the stained sections were captured using a BZ‐X700 microscope (Keyence, Osaka, Japan).

### 2.7 RT‐qPCR analysis

Coronal sections were cut to 70‐μm thickness using a cryostat (Leica 3050). Adjacent sections were stained with 0.5% thionin (Sigma‐Aldrich, St. Louis, MO, USA) for brain region identification. The brain regions were determined according to the existing literature.^48^ Samples of the hippocampus, PVN, and LS were removed using sample corers (Fine Science Tools, Heidelberg, Germany), total RNA was isolated using the ReliaPrep RNA Tissue Miniprep System (Promega, Madison, WI, USA), and cDNA was synthesized using the PrimeScript RT Reagent Kit (Takara Bio Inc., Otsu, Japan). RT‐qPCR was performed using the Thunderbird Probe qPCR mix (Toyobo Co., Ltd., Osaka, Japan) with a StepOnePlus real‐time PCR System (Applied Biosystems, Foster City, CA, USA) and PrimeTime qPCR Assays (Integrated DNA Technologies, Inc., Coralville, IA, USA): *Actb* (Mm.PT.58.33257376. gs), *Nr3c1* (Mm.PT.58.42952901), *Nr3c2* (Mm.PT.58.30752774), *Crh* (Mm.PT.58.32061593), *Arc* (Mm.PT.58.5865502.g), and *Fos* (Mm.PT.58.29977214). The ΔΔCT method was used to calculate the expression levels of the target transcripts, normalized to *Actb*, and presented as fold changes compared with B6J values at basal conditions (Basal‐B6J).

### 2.8 Statistical analysis

Statistical analyses were performed using the R statistical software (version 4.4.1; R Core Team, 2023). We employed a combination of parametric and nonparametric methods tailored to the characteristics of each dataset.

**FIGURE 1:**
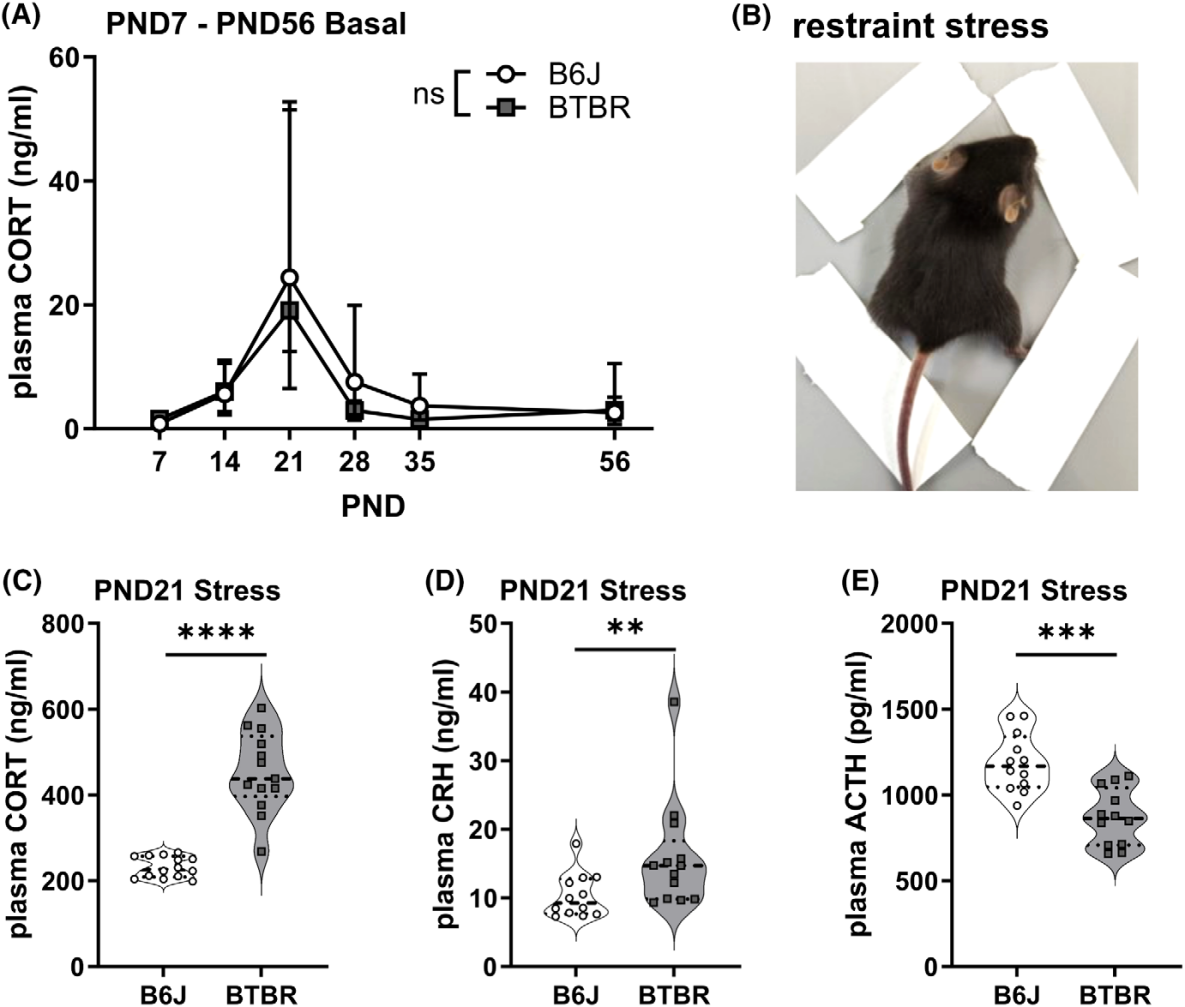
Plasma levels of stress‐related endocrine factors. (A) Developmental changes of basal corticosterone (CORT) levels from postnatal day (PND) 7 to 56 (*n* = 10–13 for each group). Data were analyzed using Aligned Rank Transform ANOVA, followed by post‐hoc tests using Holm's method for *p*‐value adjustment. Data points represent median values, and error bars indicate 95% confidence intervals. ns: Not significant between groups. Exact *p‐*values and effect sizes are reported in Tables S1 and S2. (B) An image of restraint stress on PND 21 (C–E) Plasma levels of stress‐related endocrine factors after 30 min of restraint stress on PND 21 (C) CORT levels (*n* = 14 B6J, *n* = 13 BTBR), (D) corticotropin‐releasing hormone (CRH) levels (*n* = 12 B6J, *n* = 13 BTBR), and (E) adrenocorticotropic hormone (ACTH) levels (*n* = 12 B6J, *n* = 12 BTBR). Data in C and E were analyzed using Welch's *t*‐test, while data in D were analyzed using the Brunner–Munzel test due to non‐normal distribution. Violin plots show individual data points, median (horizontal line), and probability density. **p < 0.01, ***p < 0.001, ****p < 0.0001.

To analyze the mouse group and PND effects on plasma CORT levels (Figure 1A), we implemented the Aligned Rank Transform (ART) ANOVA^49^ via the ARTool package. This method allowed us to evaluate the main effects of Group and PND as well as their interaction without assuming normality or homoscedasticity of the data. When the main effects or interactions were significant, post‐hoc tests were conducted using Holm's method for *p*‐value adjustment. Effect sizes for these analyses were reported as partial *η* ^2^, calculated using the effectsize package. For the plasma levels of stress‐related endocrine factors on PND 21 (Figure 1C–E), we first assessed the normality of distribution using the Shapiro–Wilk test. When the data were normally distributed, Welch's *t*‐test was used (Figure 1C,E). For non‐normally distributed data, we used the Brunner–Munzel test, a nonparametric method robust to heteroscedasticity^50^ (Figure 1D). Effect sizes are reported as Cohen's *d* for both parametric and nonparametric tests in this dataset. RT‐qPCR was performed using a step‐down procedure for multiple comparisons.^51^ We employed the Brunner–Munzel test for these comparisons, with the Probability of Superiority (PS) as an effect size measure. In cases where values between the Basal and Stress groups were completely separated, resulting in undefined test statistics (‐Inf) and incalculable *p*‐values for the Brunner–Munzel test, we used the Mann–Whitney *U*‐test instead, with Cliff's delta as the effect size measure. Adjusted *p*‐values were calculated for all analyses, with significance levels denoted as **** for *p* < 0.0001, *** for *p* < 0.001, ** for *p* < 0.01, * for *p* < 0.05, and ns for *p* ≥ 0.05. The Brunner–Munzel test was performed using the “lawstat” package, while Cliff's Delta was calculated using the “effsize” package. The significance level was set at *p* < 0.05 in all analyses.

The data were visualized using GraphPad Prism 10 (GraphPad Software, San Diego, California, USA).

## 3 RESULTS

### 3.1 Developmental changes in basal CORT levels and acute stress response of HPA axis on PND 21

To examine basal CORT levels during postnatal development, we analyzed plasma CORT levels on PND 7, 14, 21, 28, 35, and 56 using ELISA. In both groups, basal CORT levels showed characteristic changes, with a peak on PND 21. There was no significant main effect of the mouse group on basal CORT levels (*p* = 0.11, partial *η* ^2^ = 0.019). However, we observed a significant main effect of PND (*p* < 0.001, partial *η* ^2^ = 0.394). The interaction between the mouse group and PND was not significant (*p* = 0.248, partial *η* ^2^ = 0.049) (Figure 1A, Table S1). Post hoc pairwise comparisons of the main effects of PND revealed significant differences between several time points. Notably, CORT levels on PND 21 were significantly higher than those on PND 7 (*p* < 0.0001), PND 14 (*p* = 0.002), PND 28 (*p* = 0.0017), PND 35 (*p* < 0.0001), and PND 56 (*p* < 0.0001) (Tables S1 and S2). As previous studies have reported that adult BTBR mice show higher CORT levels than control mice after stress exposure,^22^, ^24^ we investigated the endocrine response to acute stress exposure on PND 21, the period of the highest basal CORT level (Figure 1B). The plasma CORT levels in BTBR mice after 30 min of restraint stress on PND 21 were significantly higher than those in B6J mice (*p* < 0.0001, Cohen's *d* = 3.3; Figure 1C). In addition, plasma CRH levels in BTBR mice after stress were significantly higher than those in B6J mice (*p* = 0.0056, Cohen's *d* = 0.9; Figure 1D). In contrast, the plasma ACTH levels in BTBR mice after stress were significantly lower than those in B6J mice (*p* = 0.0001, Cohen's *d* = 1.92; Figure 1E).

### 3.2 Expression patterns of *Nr3c1* and *Nr3c2* mRNA in the hippocampus and PVN

**FIGURE 2:**
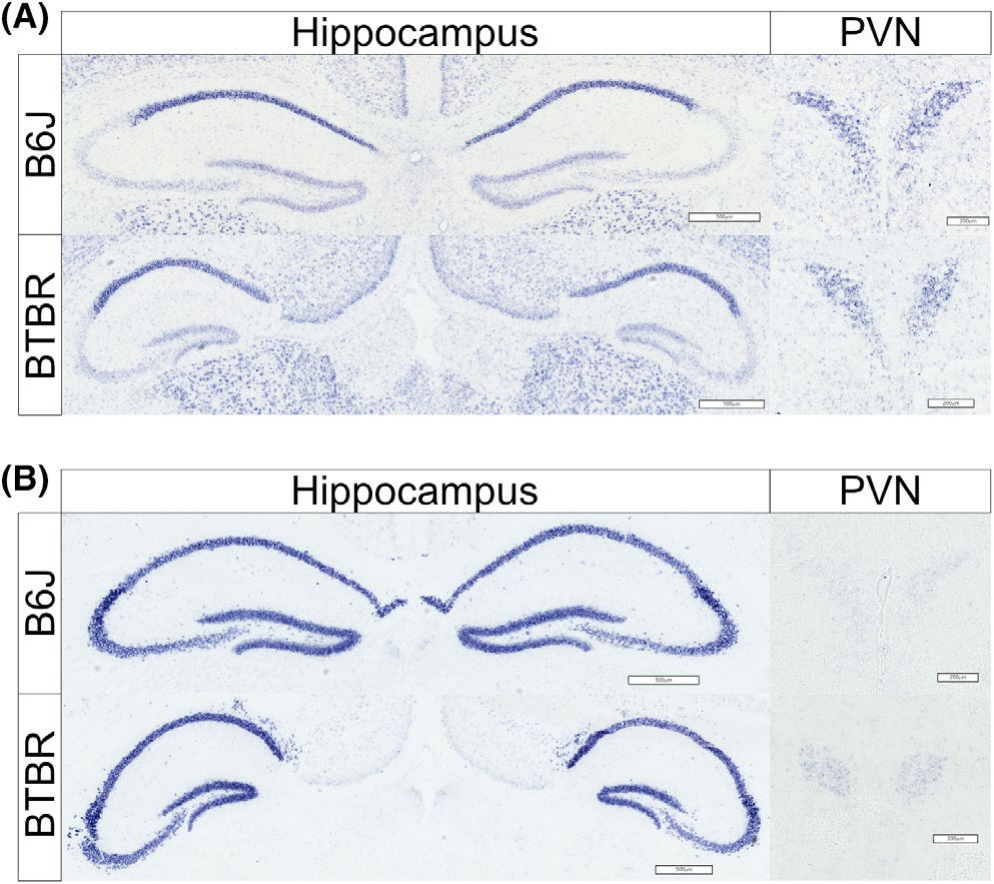
Expression patterns of glucocorticoid receptor (*Nr3c1*) and mineralocorticoid receptor (*Nr3c2*) mRNA in the hippocampus and paraventricular nucleus (PVN). (A, B) Representative images of in situ hybridization after 30 min of restraint stress at postnatal day 21 in the hippocampus (left) and (PVN, right) in B6J (top) and BTBR (bottom) mice (A) *Nr3c1* mRNA and (B) *Nr3c2* mRNA Scale bars = 500 μm (hippocampus), 200 μm (PVN).

Next, we examined the regional expression patterns of *Nr3c1* and *Nr3c2* mRNA in the PVN, which is the starting point of the HPA axis. We also examined this in the hippocampus, which regulates PVN activity on PND 21 using ISH. The hippocampus of BTBR mice was smaller than that of B6J mice and showed anatomical morphological abnormalities. However, the characteristic expression patterns of *Nr3c1* were strong in CA1 and weak in CA3 and were maintained in BTBR mice (Figure 2A, left). No remarkable anatomical differences were observed in the PVN of the BTBR mice, and *Nr3c1* mRNA expression showed a characteristic fan‐shaped staining pattern in both groups (Figure 2A, right). Furthermore, *Nr3c2* mRNA was strongly expressed in the CA1, CA3, and dentate gyrus of the hippocampus, whereas very weak expression was observed in the PVN. These expression patterns were similar in both mice (Figure 2B).

### 3.3 Expression levels of stress‐related genes and immediate early genes mRNA on PND 21 in the hippocampus

**FIGURE 3:**
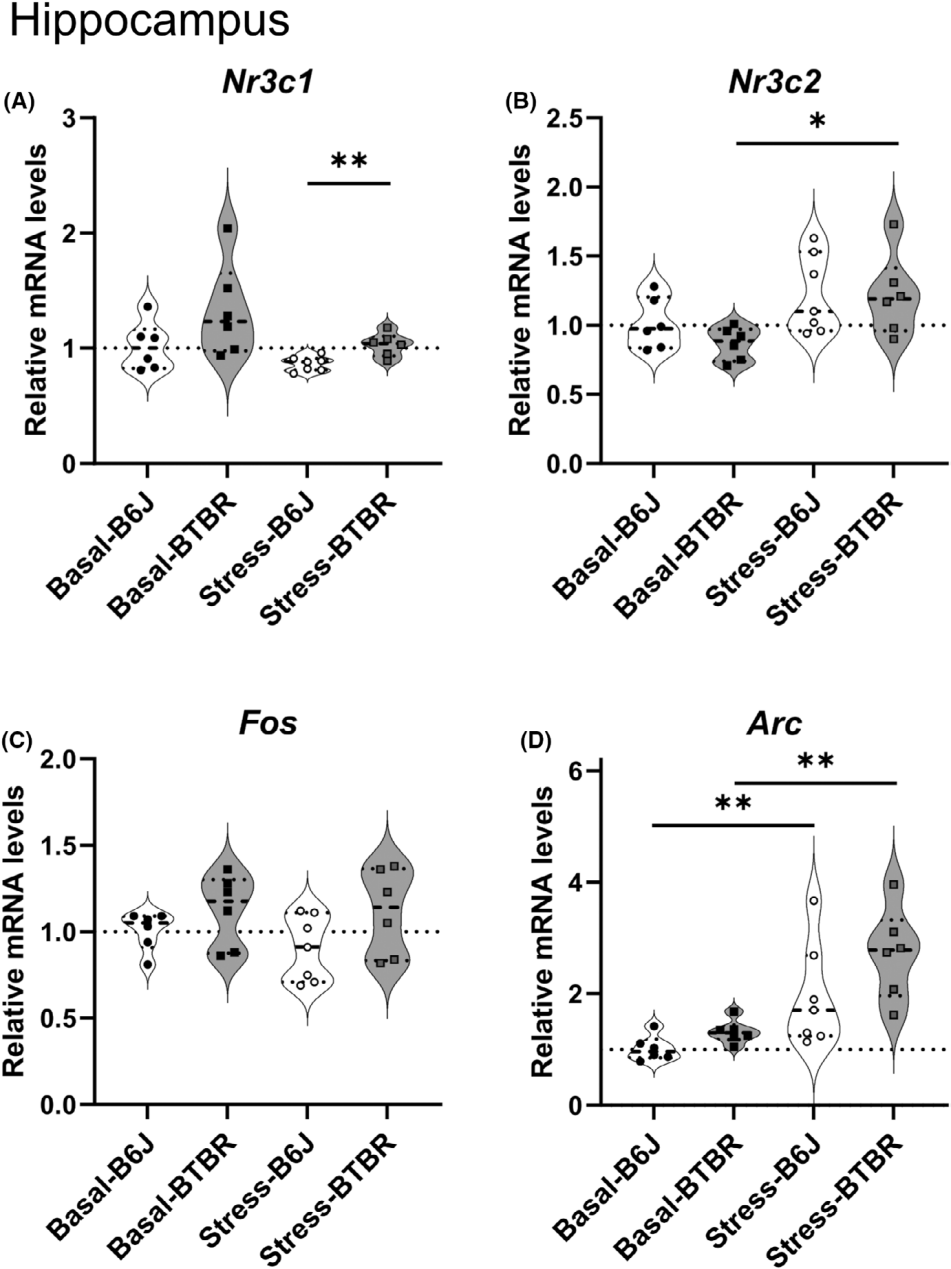
Expression of glucocorticoid receptor, mineralocorticoid receptor and immediate early genes in the hippocampus of B6J and BTBR mice under basal and stress conditions on postnatal day 21. Normalized mRNA levels of (A) *Nr3c1*, (B) *Nr3c2*, (C) *Fos*, and (D) *Arc* in the hippocampi of B6J and BTBR mice under basal conditions and 30 min after restraint stress (*n* = 6–7 per group). Expression levels were normalized to *Actb* and presented as fold changes relative to B6J‐Basal levels. Statistical analyses were performed by using a step‐down procedure for multiple comparisons. The Brunner–Munzel test was employed with Probability of Superiority as the effect size measure, except when test statistics were undefined, for which the Mann–Whitney *U*‐test was used with Cliff's Delta as the effect size measure. Violin plots show the individual data points, median (horizontal line), and probability density. Dotted lines represent B6J‐Basal expression levels. Significance levels: *p < 0.05, **p < 0.01; ns, not significant. The exact *p*‐values and effect sizes are reported in Table S3.

The expression levels of stress‐related genes (*Nr3c1* and *Nr3c2*) and immediate early genes (*Fos* and *Arc*) were quantified in the hippocampi of B6J and BTBR mice under basal conditions and acute restraint stress on PND21 (Figure 3, Table S3). *Nr3c1* mRNA expression was significantly higher in BTBR mice than in B6J mice under stress conditions (*p* = 0.0011, PS = 0.917) (Figure 3A). Under basal conditions, although not statistically significant, a moderate effect size was observed between strains (*p* = 0.17, PS = 0.778), suggesting a potential strain difference. *Nr3c2* mRNA expression significantly increased in BTBR mice following stress (*p* = 0.015, PS = 0.889) (Figure 3B). In B6J mice, although the change in *Nr3c2* expression after stress was not statistically significant, a moderate effect size was noted (*p* = 0.31; PS = 0.726), indicating a possible strain‐specific response to stress. *Fos* mRNA expression did not differ significantly across all comparisons (Figure 3C). However, comparing the strains after stress yielded a moderate‐to‐large effect size (*p* = 0.40, PS = 0.762), suggesting a potential strain difference in the stress response that may warrant further investigation. *Arc* mRNA expression increased significantly after stress in both strains, with large effect sizes (BTBR: *p* < 0.00001, PS 0.972; B6J: *p* = 0.0018, PS = 0.929) (Figure 3D). Notably, strain comparisons under both basal and stress conditions, which did not reach statistical significance, showed large effect sizes (Basal: *p* = 0.066, PS = 0.833; Stress: *p* = 0.066, PS = 0.786). These results suggest possible strain differences in *Arc* expression that may be biologically relevant despite not reaching the threshold for statistical significance.

### 3.4 Expression levels of stress‐related genes and immediate early genes mRNA on PND 21 in the PVN

**FIGURE 4:**
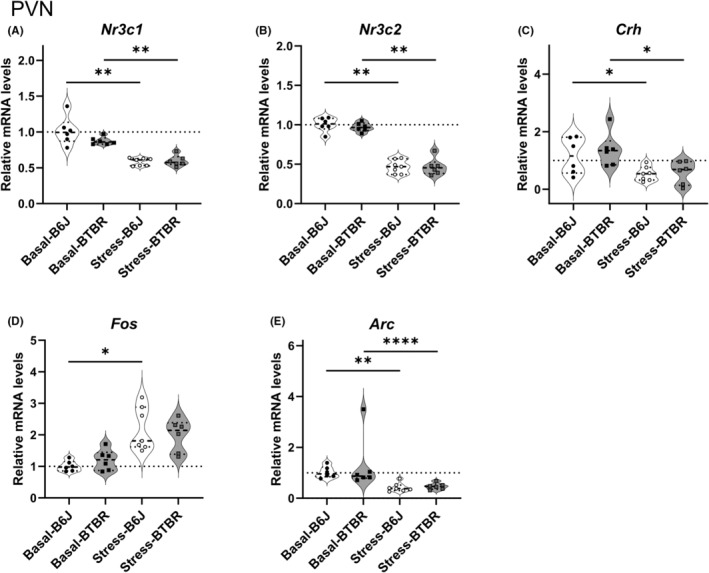
Expression of stress‐related genes in the paraventricular nucleus (PVN) of B6J and BTBR mice under basal and stress conditions on postnatal day 21. Normalized mRNA levels of (A) *Nr3c1*, (B) *Nr3c2*, (C) *Crh*, (D) *Fos*, and (E) *Arc* in the PVN of B6J and BTBR mice under basal conditions and 30 min after restraint stress (*n* = 6–7 per group). Expression levels were normalized to *Actb* and presented as fold changes relative to B6J‐Basal levels. Statistical analyses were performed by using a step‐down procedure for multiple comparisons. The Brunner–Munzel test was employed with Probability of Superiority as the effect size measure, except when test statistics were undefined, for which the Mann–Whitney *U*‐test was used with Cliff's Delta as the effect size measure. Violin plots show the individual data points, median (horizontal line), and probability density. Dotted lines represent B6J‐Basal expression levels. Significance levels: *p < 0.05, **p < 0.01, ****p < 0.0001; ns, not significant. The exact *p*‐values and effect sizes are reported in Table S3.

Next, we quantified the expression of *Nr3c1*, *Nr3c2*, *Crh*, *Fos*, and *Arc* in both groups under basal conditions and acute restraint stress in the PVN (Figure 4; Table S3) on PND21. *Nr3c1* mRNA expression levels in the PVN showed significant stress‐induced decreases in both strains with maximum effect sizes (B6J: *p* = 0.0098, Cliff's Delta = 1; BTBR: *p* = 0.0098, Cliff's Delta = 1) (Figure 4A). Under basal conditions, a small‐to‐moderate effect size was observed between the strains (*p* = 0.17, Cliff's Delta = 0.556), suggesting a potential strain difference despite the lack of statistical significance. *Nr3c2* mRNA expression levels showed similar stress‐induced changes, with significant decreases and maximum effect sizes in both strains (B6J: *p* = 0.01, Cliff's Delta = 1; BTBR: *p* = 0.01, Cliff's Delta = 1) (Figure 4B). Basal expression levels were comparable between the strains, with a small effect size (*p* = 0.50, Cliff's Delta = 0.333). *Crh* mRNA expression levels showed statistically significant stress‐induced decreases in both strains, although the effect sizes were small (BTBR, *p* = 0.012, PS 0.111; B6J, *p* = 0.037, PS 0.167) (Figure 4C). No significant differences in strain were observed under basal or stress conditions. *Fos* mRNA expression levels increased significantly after stress in B6J mice with a very large effect size (*p* = 0.014, Cliff's Delta = −1) (Figure 4D). BTBR mice showed a similar trend, with a large effect size, although the difference was not statistically significant (*p* = 0.061, Cliff's Delta = −0.778). Under basal conditions, a moderate effect size was observed between strains (*p* = 0.45, Cliff's Delta = −0.361), suggesting potential subtle strain differences. *Arc* mRNA expression levels decreased significantly after stress in both strains, with very large to maximum effect sizes (B6J: *p* = 0.010, Cliff's Delta = 0.976; BTBR: *p* = 0.010, Cliff's Delta = 1) (Figure 4E). Basal expression levels were comparable between the strains, with a small effect size (*p* = 0.69, Cliff's Delta = 0.167).

These PVN qPCR results strongly suggest gene expression suppression due to negative feedback in response to stress in both B6J and BTBR mouse strains. Unlike the hippocampus, no significant strain differences were observed in the PVN under acute stress conditions.

### 3.5 LS aberrations in BTBR mice

**FIGURE 5:**
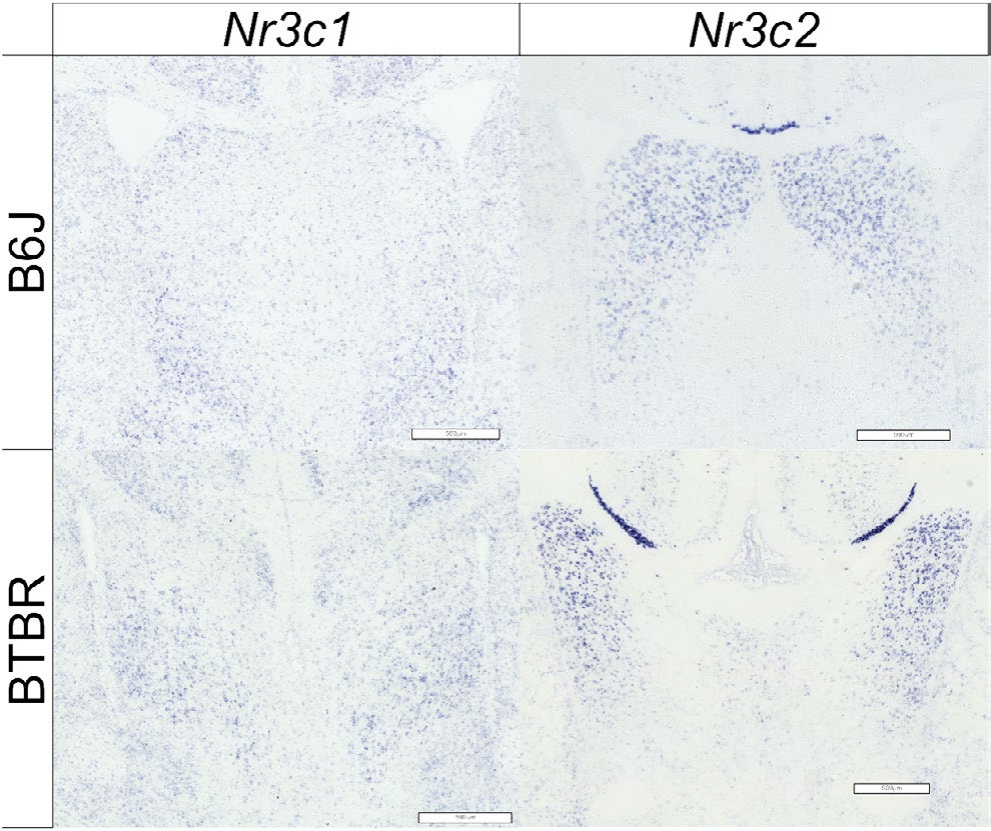
Expression patterns of glucocorticoid receptor (*Nr3c1*) and mineralocorticoid receptor (*Nr3c2*) mRNA in the lateral septum (LS). Representative images of in situ hybridization 30 min after restraint stress on postnatal day 21 in the LS of B6J (top) and BTBR mice (bottom). *Nr3c1* (left) and (B) *Nr3c2* (right) mRNA. Scale bars = 500 μm.

As BTBR mice exhibit abnormal anatomical characteristics,^46^ we examined the expression patterns and levels of *Nr3c1* and *Nr3c2* mRNA in the LS. In BTBR mice, the septum protruded into the ventricles; expression of *Nr3c1* mRNA was sparse throughout the septum, whereas strong expression of *Nr3c2* mRNA was observed on the lateral side (where the ventricles were in B6J) (Figure 5). Nissl staining revealed atypical LS morphology in BTBR mice (Figures S1 and S2).

**FIGURE 6:**
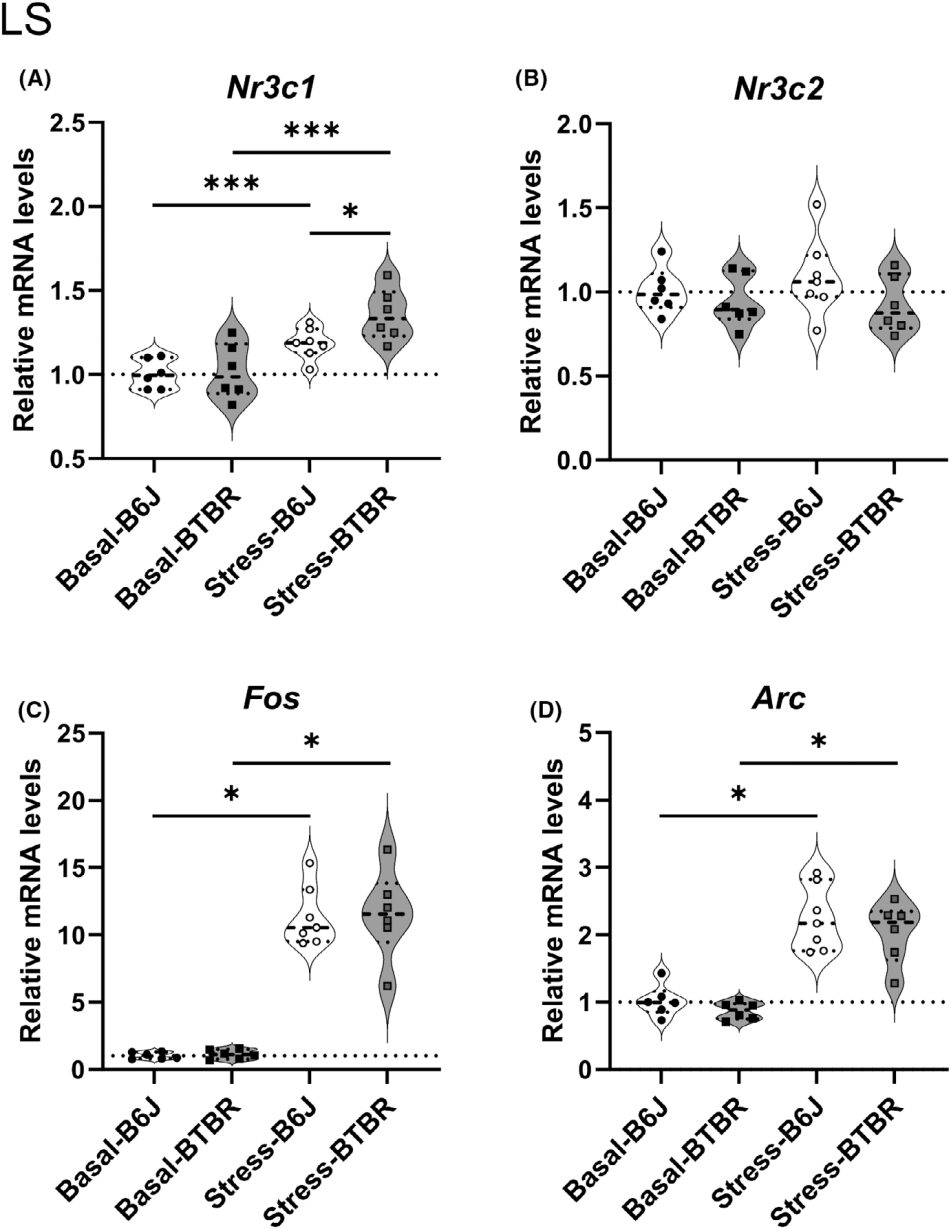
Expression of glucocorticoid receptor, mineralocorticoid receptor and immediate early genes in the lateral septum (LS) of B6J and BTBR mice under basal and stress conditions on postnatal day 21. Normalized mRNA levels of (A) *Nr3c1*, (B) *Nr3c2*, (C) *Fos*, and (D) *Arc* in the LS of B6J and BTBR mice under basal conditions and 30 min after restraint stress (*n* = 6–7 per group). Expression levels were normalized to *Actb* and presented as fold changes relative to B6J‐Basal levels. Statistical analyses were performed by using a step‐down procedure for multiple comparisons. The Brunner–Munzel test was employed with Probability of Superiority as the effect size measure, except when test statistics were undefined, for which the Mann–Whitney *U*‐test was used with Cliff's Delta as the effect size measure. Violin plots show the individual data points, median (horizontal line), and probability density. Dotted lines represent B6J‐Basal expression levels. Significance levels: *p < 0.05, ***p < 0.001; ns, not significant. The exact *p*‐values and effect sizes are reported in Table S3.

Finally, we quantified the expression levels of *Nr3c1*, *Nr3c2*, *Fos*, and *Arc* in the LS (Figure 6, Table S3) on PND21. *Nr3c1* mRNA expression levels in the LS showed significant stress‐induced increases in both strains with very large effects (BTBR: *p* = 0.000025, PS = 0.958; B6J: *p* = 0.000025, PS = 0.952). Under basal conditions, expression levels were comparable between the strains (*p* = 0.89, PS = 0.528). After stress exposure, BTBR mice exhibited significantly higher *Nr3c1* expression than B6J mice (*p* = 0.040, PS = 0.821), consistent with the pattern observed in the hippocampus.

*Nr3c2* mRNA expression levels did not show significant stress‐induced changes in either strain, unlike the significant decrease observed in the PVN. No significant differences in strain were observed under basal or stress conditions. *Fos* mRNA expression increased significantly after stress in both strains, with maximum effect sizes (B6J: *p* = 0.010, Cliff's Delta = −1; BTBR: *p* = 0.010, Cliff's Delta = −1). No significant differences in strain were observed under basal or stress conditions. *Arc* mRNA expression levels also showed significant stress‐induced increases in both strains with maximum effect sizes (B6J: *p* = 0.0101, Cliff's Delta = −1; BTBR: *p* = 0.0101, Cliff's Delta = −1). Under basal conditions, a moderate effect size was observed between the strains (*p* = 0.3064, Cliff's Delta = 0.4444), although this difference was not statistically significant.

## 4 DISCUSSION

In this study, we investigated the function of the HPA‐axis during postnatal development in BTBR mice, a mouse model of ASD. We first found that Basal CORT levels showed characteristic changes, with a peak on PND 21 (Figure 1A), consistent with previous reports in Sprague–Dawley rats, followed by a decrease in basal CORT levels on PND 56. However, this result contradicts a previous study that reported an increase in basal CORT levels from 3 to 8 weeks of age in BALB/c mice. This inconsistency may reflect differences in the mouse strains used, as BALB/c mice are more reactive to stressors than C57BL/6 mice and exhibit greater HPA hormonal alterations and behavioral disturbances.^52^ Nevertheless, numerous reports on the stress hyporesponsive period during PND 2–14 show a reduction in the stress response, such as altered glucocorticoid secretion, after stress exposure during the postnatal stages.^53^ However, few studies have focused on basal CORT level changes during early life stages; therefore, the physiological significance of basal CORT level changes during postnatal development remains largely unknown. It is possible that changes in CORT levels during postnatal developmental stages regulate mechanisms distinct from the stress response, such as neural circuit development.^54^, ^55^

In addition, we found no significant difference between CORT level changes in B6J and BTBR mice (Figure 1A), suggesting that the mechanisms that create basal CORT level changes during the postnatal developmental stages in BTBR mice might be intact. Furthermore, the circadian rhythm affects basal CORT levels, although this was not examined in the present study. Although the mechanisms underlying the circadian rhythm of basal CORT levels in BTBR mice are unknown, we have previously reported that BTBR mice maintain a normal circadian rhythm for behavioral activity.^20^

Previous studies have reported that adult BTBR mice exhibit higher CORT levels than control mice after stress exposure.^22^, ^24^ We found that the CORT and CRH levels in BTBR mice after 30 min of restraint stress on PND 21 were significantly higher than those in B6J mice (Figure 1C,D), suggesting that the abnormal stress response in BTBR mice was already present at this age. Unexpectedly, the ACTH levels in BTBR mice were lower than those in B6J mice (Figure 1E). Osterlund et al. reported that restraint stress rapidly induces a large increase in plasma ACTH levels, with a rapid decline within 30 min, resulting in a faster attenuation of ACTH levels than CORT in rats.^56^ Additionally, a stronger attenuation of ACTH levels after stress is induced by higher CORT levels.^56^ In the present study, it is possible that stronger negative feedback on ACTH levels was induced at this time because of the higher CORT levels in BTBR mice, resulting in lower ACTH levels in BTBR mice than in B6J mice.

BTBR mice lack the corpus callosum and have severely reduced hippocampal commissures.^34^ We did not detect a marked change in the regional expression patterns of *Nr3c1* or *Nr3c2* mRNA in the hippocampus of BTBR mice using in situ hybridization (Figure 2). In contrast, RT‐qPCR revealed two key findings regarding hippocampal glucocorticoid receptor gene expression. First, while *Nr3c1* mRNA levels did not change with stress in either strain, BTBR mice showed higher *Nr3c1* expression than B6J mice under stress conditions (Figure 3A), consistent with previous reports. Second, *Nr3c2* mRNA expression increased significantly after stress only in BTBR mice (Figure 3B). This distinct pattern suggested an altered balance between *Nr3c1* and *Nr3c2* in stressed BTBR mice. These observations suggest that the hippocampi of BTBR mice are dysfunctional, indicating that the negative feedback mechanism from the hippocampus to the HPA axis may not function properly. This hippocampal dysfunction may potentially contribute to the aberrant stress response and ASD‐like behaviors observed in BTBR mice. Additionally, RT‐qPCR revealed patterns of immediate‐early gene expression. *Fos* mRNA expression did not show statistically significant differences between strains or in response to stress immediately after the 30‐min restraint period (Figure 3C). In contrast, *Arc* mRNA expression increased significantly after stress in both BTBR and B6J mice, with no significant strain differences (Figure 3D). These findings suggest that both strains exhibited similar immediate early gene responses to acute restraint stress in the hippocampus; *Arc* showed a detectable increase in expression at this point, whereas *Fos* did not. In the PVN, we did not detect changes in the morphological or regional expression patterns of *Nr3c1* and *Nr3c2* mRNA using in situ hybridization in BTBR mice. However, our RT‐qPCR results showed stress‐induced decreases in *Nr3c1* and *Nr3c2* expression in both strains (Figure 4A,B), suggesting a robust negative feedback mechanism in response to stress. Interestingly, unlike in the hippocampus, no significant strain differences were observed in the PVN under acute stress conditions. These findings suggest that the abnormal stress response in BTBR mice might primarily originate from alterations in hippocampal function rather than from direct changes in the PVN. However, these results should be interpreted with caution. A previous study reported that stress induces lower *Fos* expression in the PVN of BTBR mice compared to that in B6J mice,^35^ which appears to contradict our observations. This discrepancy may be attributed to differences in the stress exposure methods, evaluation techniques (mRNA vs. protein), and sampling time points. Given these conflicting results, we cannot conclusively state that the PVN functions normally in BTBR mice based on our findings alone. The stress response in the PVN of the BTBR mice may involve subtle or complex alterations that were not captured in the current experimental design. Further investigations, potentially involving a wider range of time points and both mRNA and protein analyses, are needed to fully elucidate the stress response mechanisms in the PVN of BTBR mice.

Consistent with previous reports,^46^ we found that the LS of the BTBR mice exhibited abnormal anatomical characteristics (Figure 5). In addition, we observed higher expression levels of *Nr3c1* mRNA by RT‐qPCR (Figure 6D) and comparable levels of *Nr3c2* mRNA (Figure 6E) after stress in the LS of BTBR mice than in B6J mice. This pattern differs from our observations in the hippocampus, suggesting region‐specific alterations in glucocorticoid receptor expression. Increased *Nr3c1* expression without a corresponding change in *Nr3c2* in the LS indicates a potential imbalance in glucocorticoid signaling, specifically in this region, which may contribute to altered stress responses in BTBR mice. The LS plays a crucial role in various neuropsychiatric conditions, including anxiety, depression, eating disorders, and comorbidities.^40^ It receives inputs from the hippocampus and projects inhibitory neurons to the medial hypothalamus, which projects inhibitory neurons to the PVN.^38^ This circuitry suggests LS abnormalities influence the stress response by modulating PVN activity. Furthermore, the LS has been reported to play an important role in social behavior.^39^ Given the involvement of the LS in social behavior and its connections with stress‐responsive regions, such as the hippocampus and PVN, the abnormalities observed in the LS may be involved in the autistic‐like behavior characteristic of BTBR mice. Further investigations into how these LS alterations affect downstream signaling and behavior could provide valuable insights into the neurobiological basis of autism‐like phenotypes in this mouse model.

Our study focused on characterizing the developmental trajectory of the HPA axis in BTBR mice without pharmacological intervention. Although this approach provides valuable insights into the natural progression of HPA axis function in this ASD model, it does not directly manipulate the system. Future studies employing pharmacological approaches, such as corticosterone synthesis inhibitors or glucocorticoid receptor antagonists, could further elucidate the role of HPA axis dysregulation in the BTBR phenotype and potentially identify therapeutic targets.

This study was conducted only on male mice, partly because of the higher incidence of ASD in males. However, given the potential sex differences in stress responses and ASD‐related behaviors, future research should include female mice to provide a more comprehensive understanding of stress responses in the BTBR ASD model.

## 5 CONCLUSIONS

We investigated basal CORT levels during postnatal developmental stages in mice and demonstrated characteristic basal CORT changes, with a peak on PND 21. Our study of stress responses in BTBR mice on PND 21 revealed higher CORT and CRH levels but lower ACTH levels compared to those in B6J mice after acute stress exposure. We observed region‐specific alterations in glucocorticoid receptor expression. In the hippocampus, *Nr3c1* mRNA levels were significantly higher in BTBR mice than in B6J mice under stressful conditions. *Nr3c2* mRNA expression was significantly increased in BTBR mice following stress, whereas B6J mice showed a moderate, nonsignificant increase. In the PVN, both strains exhibited a significant stress‐induced decrease in *Nr3c1* and *Nr3c2* mRNA expression, suggesting a negative feedback mechanism. In the LS, BTBR mice exhibited higher *Nr3c1* mRNA expression after stress than B6J mice, whereas *Nr3c2* levels were comparable between the two strains. In conclusion, these results suggest that BTBR mice exhibit disrupted region‐specific alterations in stress responses on PND 21.

## AUTHOR CONTRIBUTIONS

N.E. and N.M. conceived of and designed the study. N.E., A.T., S.G., H. N., T.M., N.H., M. K., and M.O. conducted the experiments and analyzed the data. M.M. provided mice. N.E. and N.M. prepared the manuscript. All the authors have read and approved the final version of the manuscript.

## FUNDING INFORMATION

This study was supported in part by Grants‐in‐Aid for Scientific Research from the Japan Society for the Promotion of Science (17H07032, 19 K16899, and 22 K15754 to N.E.; 17H06060, 17 K19922, and 19H03539 to M.N.). The funder was not involved in the study design, data collection, analysis, publication decision, or manuscript preparation.

## CONFLICT OF INTEREST STATEMENT

The authors declare no conflict of interest.

## ETHICS STATEMENT

Approval of the Research Protocol by an Institutional Reviewer Board: N/A.

Informed Consent: N/A.

Registry and the Registration No. of the Study/Trial: N/A.

Animal Studies: All animal experimental protocols used in this study were approved by the Animal Care Committee of Nara Medical University. The study was conducted following local legislation and institutional requirements.

## Supporting information


**Data S1:** Supporting Information.

**Data S2:** Supporting Information.

## Data Availability

The data supporting the findings of this study are available in Supplementary—Data S2.
