# Supplementary material for: Dysregulated HPA axis during postnatal developmental stages in the BTBR T+ Itpr3tf/J mouse: A model of autism spectrum disorder
Source: Neuropsychopharmacol Rep. 2024 Nov 28;45(1):e12508. doi: 10.1002/npr2.12508 (PMC11660766; doi:10.1002/npr2.12508)
Supplement: Supplementary file 1 — Data S1: Supporting Information. [file NPR2-45-e12508-s001.pdf]

Term	Df	Df.res	Sum Sq	Sum Sq.res	F value	p value	partial η²
Mouse group	1	132	4096.14	210349.58	2.57	0.111	0.019
PND	5	132	87112.43	133907.64	17.17	< 0.001	0.394
Mouse group:PND	5	132	10110.45	198094.97	1.35	0.248	0.049

Supplementary Table 1: Results of Aligned Rank Transform ANOVA for the experiment in Fig 1A

Note: Df = degrees of freedom; Df.res = residual degrees of freedom; Sum Sq = Sum of Squares; Sum Sq.res = residual Sum of Squares

Effect sizes for these analyses were reported as partial η^2.

contrast	estimate	SE	df	t.ratio	p.value
PND7 - PND14	-47.008	9.401	132	-5.000	2.05E-05
PND7 - PND21	-81.578	9.314	132	-8.758	1.25E-13
PND7 - PND28	-46.668	9.314	132	-5.010	2.05E-05
PND7 - PND35	-26.976	9.314	132	-2.896	0.0310
PND7 - PND56	-29.991	9.538	132	-3.144	0.0165
PND14 - PND21	-34.571	9.106	132	-3.797	0.0020
PND14 - PND28	0.340	9.106	132	0.037	1
PND14 - PND35	20.032	9.106	132	2.200	0.1773
PND14 - PND56	17.017	9.335	132	1.823	0.2823
PND21 - PND28	34.910	9.016	132	3.872	0.0017
PND21 - PND35	54.603	9.016	132	6.056	1.90E-07
PND21 - PND56	51.587	9.247	132	5.579	1.71E-06
PND28 - PND35	19.692	9.016	132	2.184	0.1773
PND28 - PND56	16.677	9.247	132	1.803	0.2823
PND35 - PND56	-3.015	9.247	132	-0.326	1

Supplementary Table 2: Post-hoc Pairwise Comparisons for the Main Effect of PND (Follow-up to Supplementary Table 1)

Note: SE = Standard Error

The *P*-values were adjusted for multiple comparisons using Holm's method.

Region	Gene	rank	comparison	adjusted p value	effect size	effect size type	significant
Hippocampus	Nr3c1	1	Stress-B6J vs Stress-BTBR	0.00110	0.917	PS	**
		2	Basal-B6J vs Basal-BTBR	0.17016	0.778	PS	ns
		3	Basal-B6J vs Stress-B6J	0.18855	0.262	PS	ns
		4	Basal-BTBR vs Stress-BTBR	0.18855	0.25	PS	ns
	Nr3c2	1	Basal-BTBR vs Stress-BTBR	0.01493	0.889	PS	*
		2	Basal-B6J vs Stress-B6J	0.30846	0.726	PS	ns
		3	Basal-B6J vs Basal-BTBR	0.30846	0.292	PS	ns
		4	Stress-B6J vs Stress-BTBR	1	0.5	PS	ns
	Fos	1	Stress-B6J vs Stress-BTBR	0.40305	0.762	PS	ns
		2	Basal-B6J vs Basal-BTBR	0.54653	0.722	PS	ns
		3	Basal-B6J vs Stress-B6J	0.60171	0.357	PS	ns
		4	Basal-BTBR vs Stress-BTBR	0.88914	0.472	PS	ns
	Arc	1	Basal-BTBR vs Stress-BTBR	0.00000	0.972	PS	****
		2	Basal-B6J vs Stress-B6J	0.00181	0.929	PS	**
		3	Basal-B6J vs Basal-BTBR	0.06577	0.833	PS	ns
		4	Stress-B6J vs Stress-BTBR	0.06577	0.786	PS	ns
PVN	Nr3c1	1	Basal-B6J vs Stress-B6J	0.00984	1	Cliff's Delta	**
		2	Basal-BTBR vs Stress-BTBR	0.00984	1	Cliff's Delta	**
		3	Basal-B6J vs Basal-BTBR	0.16827	0.556	Cliff's Delta	ns
		4	Stress-B6J vs Stress-BTBR	1	-0.024	Cliff's Delta	ns
	Nr3c2	1	Basal-B6J vs Stress-B6J	0.01000	1	Cliff's Delta	**
		2	Basal-BTBR vs Stress-BTBR	0.01000	1	Cliff's Delta	**
		3	Basal-B6J vs Basal-BTBR	0.50016	0.333	Cliff's Delta	ns
		4	Stress-B6J vs Stress-BTBR	0.88594	0.071	Cliff's Delta	ns
	Crh	1	Basal-BTBR vs Stress-BTBR	0.01159	0.111	PS	*
		2	Basal-B6J vs Stress-B6J	0.03706	0.167	PS	*
		3	Basal-B6J vs Basal-BTBR	0.73035	0.583	PS	ns
		4	Stress-B6J vs Stress-BTBR	0.73035	0.571	PS	ns
	Fos	1	Basal-B6J vs Stress-B6J	0.01362	-1	Cliff's Delta	*
		2	Basal-BTBR vs Stress-BTBR	0.06128	-0.778	Cliff's Delta	ns
		3	Basal-B6J vs Basal-BTBR	0.44776	-0.361	Cliff's Delta	ns
		4	Stress-B6J vs Stress-BTBR	0.56718	0.214	Cliff's Delta	ns
	Arc	1	Basal-B6J vs Stress-B6J	0.01000	0.976	Cliff's Delta	**
		2	Basal-BTBR vs Stress-BTBR	0.01000	1	Cliff's Delta	**
		3	Stress-B6J vs Stress-BTBR	0.63259	-0.262	Cliff's Delta	ns
		4	Basal-B6J vs Basal-BTBR	0.68840	0.167	Cliff's Delta	ns
LS	Nr3c1	1	Basal-BTBR vs Stress-BTBR	0.0000248	0.958	PS	***
		2	Basal-B6J vs Stress-B6J	0.0000248	0.952	PS	***
		3	Stress-B6J vs Stress-BTBR	0.04007	0.821	PS	*
		4	Basal-B6J vs Basal-BTBR	0.88822	0.528	PS	ns
	Nr3c2	1	Stress-B6J vs Stress-BTBR	0.67495	0.286	PS	ns
		2	Basal-B6J vs Basal-BTBR	0.67495	0.361	PS	ns
		3	Basal-B6J vs Stress-B6J	0.67495	0.619	PS	ns
		4	Basal-BTBR vs Stress-BTBR	0.77607	0.444	PS	ns
	Fos	1	Basal-B6J vs Stress-B6J	0.01015	-1	Cliff's Delta	*
		2	Basal-BTBR vs Stress-BTBR	0.01015	-1	Cliff's Delta	*
		3	Stress-B6J vs Stress-BTBR	0.81018	-0.167	Cliff's Delta	ns
		4	Basal-B6J vs Basal-BTBR	0.81018	-0.111	Cliff's Delta	ns
	Arc	1	Basal-B6J vs Stress-B6J	0.01015	-1	Cliff's Delta	*
		2	Basal-BTBR vs Stress-BTBR	0.01015	-1	Cliff's Delta	*
		3	Basal-B6J vs Basal-BTBR	0.30636	0.444	Cliff's Delta	ns
		4	Stress-B6J vs Stress-BTBR	0.56718	0.214	Cliff's Delta	ns

Supplementary Table 3: RT-qPCR Gene Expression Analysis Results with Step-down Multiple Comparison Procedure in Fig 3, 4, 6

Note: Genes were ranked based on their *P*-values, with rank 1 assigned the lowest *P*-value. *P*-values were adjusted for multiple comparisons by using a stepwise procedure. The Brunner–Munzel test was used for comparisons with the Probability of Superiority (PS) as the effect size. The Mann–Whitney U test with Cliff's delta was used when the Brunner–Munzel test statistics were undefined. Significance levels: **** $P < 0.0001$, *** $P < 0.001$, ** $P < 0.01$, * $P < 0.05$, ns $P \geq 0.05$. PS = Probability of Superiority.

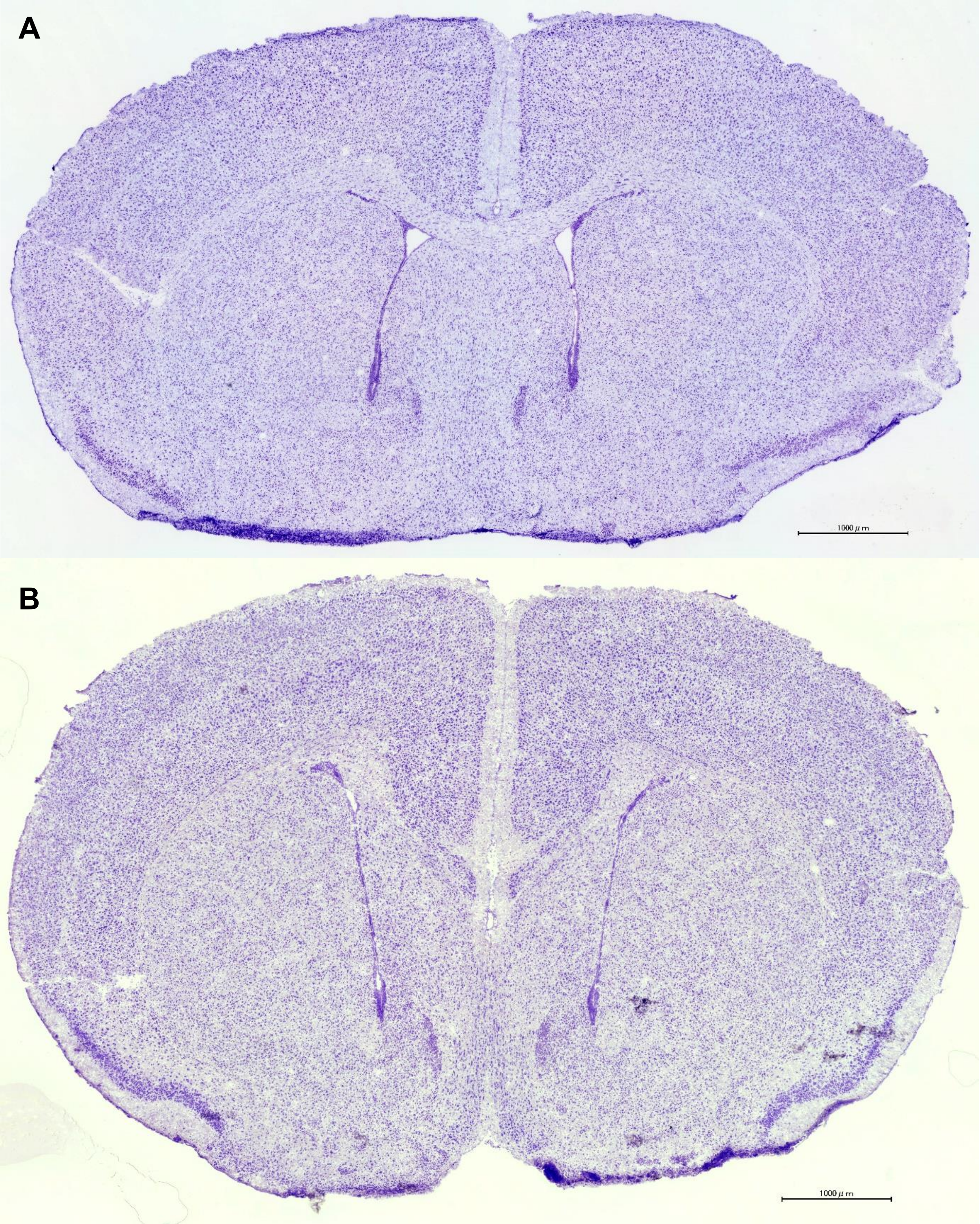

Supplementary Fig. 1

Representative images of the Nissl stain section, including LS on PND 21 (A) B6J (B) BTBR Scale bars = 1000 μm.

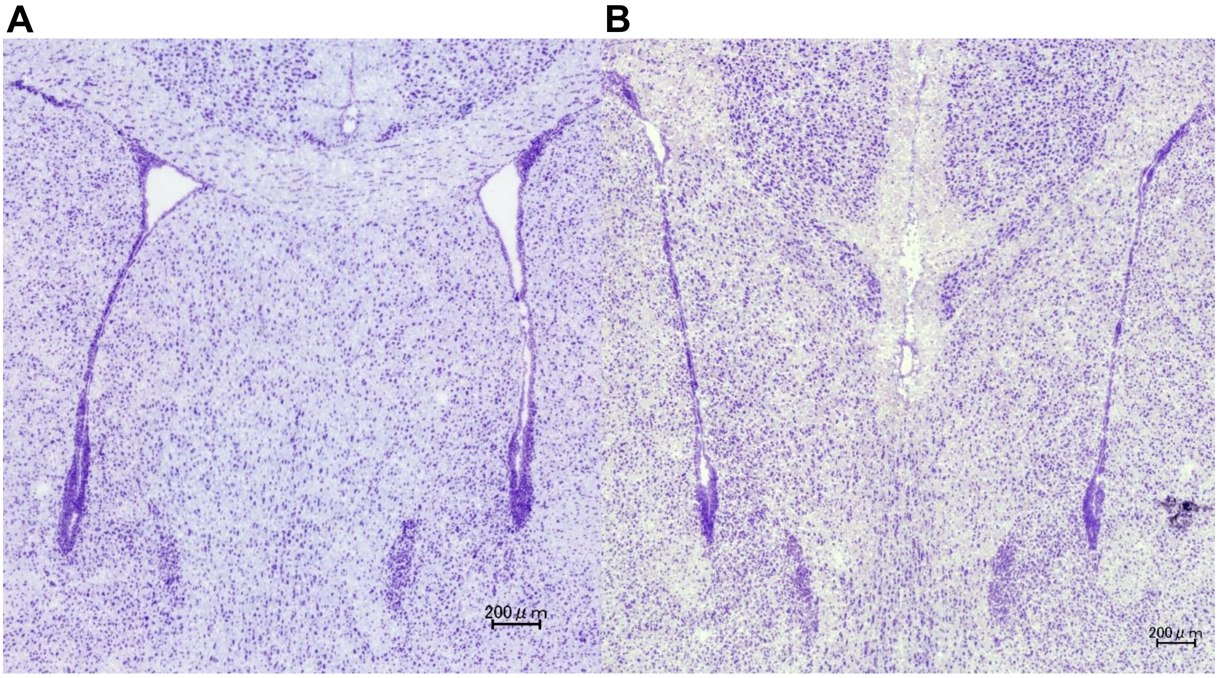

Supplementary Fig. 2

Representative images of Nissl stain in LS on PND 21 (A) B6J (B) BTBR

Scale bars = 200 μm.
